# The Impact of Electrophysiological Diversity on Pattern Completion in Lithium Nonresponsive Bipolar Disorder: A Computational Modeling Approach

**DOI:** 10.1002/brb3.70209

**Published:** 2025-01-20

**Authors:** Abraham Nunes, Selena Singh, Anouar Khayachi, Shani Stern, Thomas Trappenberg, Martin Alda

**Affiliations:** ^1^ Department of Psychiatry Dalhousie University Halifax Nova Scotia Canada; ^2^ Faculty of Computer Science Dalhousie University Halifax Nova Scotia Canada; ^3^ Department of Psychology, Neuroscience & Behaviour McMaster University Hamilton Ontario Canada; ^4^ Montreal Neurological Institute, Department of Neurology & Neurosurgery McGill University Montreal Quebec Canada; ^5^ Sagol Department of Neurobiology, Faculty of Natural Sciences University of Haifa Haifa Israel

**Keywords:** bipolar disorder, CA3, computational modeling, lithium

## Abstract

**Introduction:**

Patients with bipolar disorder (BD) demonstrate episodic memory deficits, which may be hippocampal‐dependent and may be attenuated in lithium responders. Induced pluripotent stem cell–derived CA3 pyramidal cell–like neurons show significant hyperexcitability in lithium‐responsive BD patients, while lithium nonresponders show marked variance in hyperexcitability. We hypothesize that this variable excitability will impair episodic memory recall, as assessed by cued retrieval (pattern completion) within a computational model of the hippocampal CA3.

**Methods:**

We simulated pattern completion tasks using a computational model of the CA3 with different degrees of pyramidal cell excitability variance. Since pyramidal cell excitability variance naturally leads to a mix of hyperexcitability and hypoexcitability, we also examined what fraction (hyper‐ vs. hypoexcitable) was predominantly responsible for pattern completion errors in our model.

**Results:**

Pyramidal cell excitability variance impaired pattern completion (linear model *β* = −2.00, *SE* = 0.03, *p* < 0.001). The effect was invariant to all other parameter settings in the model. Excitability variance, specifically hyperexcitability, increased the number of spuriously active neurons, increasing false alarm rates and producing pattern completion deficits. Excessive inhibition also induces pattern completion deficits by limiting the number of correctly active neurons during pattern retrieval.

**Conclusions:**

Excitability variance in CA3 pyramidal cell–like neurons observed in lithium nonresponders may predict pattern completion deficits in these patients. These cognitive deficits may not be fully corrected by medications that minimize excitability. Future studies should test our predictions by examining behavioral correlates of pattern completion in lithium‐responsive and ‐nonresponsive BD patients.

## Graphical abstract

Diverse CA3 pyramidal cell excitability found in lithium‐nonresponsive bipolar disorder is predicted by a computational model to impair CA3 pattern completion.

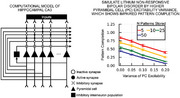

## 1 Introduction

Bipolar disorder (BD) is a chronic and debilitating mental illness with unknown neurobiology (American Psychiatric Association 2013), characterized by episodes of mania and depression (Grande et al. 2015). Lithium mitigates symptoms for many patients, but approximately two‐thirds remain nonresponsive (Garnham et al. 2007). Lithium nonresponders may have poor episodic memory compared to patients who are stable on lithium monotherapy (Burdick et al. 2020), raising questions about the neural mechanisms of episodic memory in BD and treatment responsiveness.

The hippocampal CA3 region is known for its role in memory processing, particularly in rapid one‐shot learning (Nakashiba et al. 2008) and pattern completion (Neunuebel and Knierim 2014; Nakazawa et al. 2002; Gold and Kesner 2005), which is the ability to retrieve a complete representation from any of that memory's parts (Rolls 2013). Pattern completion is supported by the extensive recurrent collateral connections in CA3 (Marr 1971; Bennett, Gibson, and Robinson 1994), which cause the CA3 to function as an *autoassociative attractor network* (Marr 1971; McNaughton and Morris 1987). Pattern completion is believed to be disrupted in psychiatric conditions such as schizophrenia, which may have a role in the formation of delusions (Tamminga, Stan, and Wagner 2010; Neymotin et al. 2011; Tamminga et al. 2012). Given the genetic relatedness of BD and schizophrenia (Anttila et al. 2018) and BD's association with both psychosis (Goes et al. 2007) and declarative memory impairments (Burdick et al. 2020; Cardenas et al. 2016; Bora 2018; Keramatian, Torres, and Yatham 2021; Nitzburg et al. 2017), it is plausible that similar hypothesized pattern completion deficits may be found in BD. Several lines of research also suggest that CA3 structure, function, and electrophysiology may be impaired in BD, which we review below.

First, many genetic variants associated with BD and lithium responsiveness are also implicated in CA3 structure and function. The GRIN1 gene, which encodes the NR1 subunit of the *N*‐methyl‐d‐aspartate receptor (NMDAR), is associated with and downregulated in BD (Mundo et al. 2003; Bundo et al. 2021) (but see Georgi et al. 2006) and schizophrenia (Catts et al. 2016). Animal models demonstrate that GRIN1 mediates the integrity of conjunctive and associative representations in the CA3 (Nakazawa et al. 2002; McHugh and Tonegawa 2009). In addition to the BDNF‐NTRK2 pathway being associated with BD (Bundo et al. 2021), completed suicide (Ernst et al. 2009), and response to mood stabilizers (Li et al. 2011; Hashimoto 2020; Gideons et al. 2017; Wang et al. 2013) at the behavioral level, its involvement extends down to the cellular and circuit level as well, regulating dendritic spine density in CA3 (Bennett and Lagopoulos 2014) and the establishment of functional circuitry between the dentate gyrus and CA3 (Szymanski and Minichiello 2022). Although these genetic abnormalities may also predispose broader neurobiological changes in BD, their overlap with CA3 structure and functioning suggests that studying this brain region in BD is an important research direction.

In addition to genetic abnormalities, functional impairment may be partly attributable to reduced hippocampal volume in BD. The largest analysis of hippocampal subfield volumes in BD (1472 patients and 3226 controls) has found significantly smaller CA3 volume in BD patients (Cohen's *d* = −0.20) (Haukvik et al. 2022): an abnormality which may be associated with impaired memory recall (Chadwick, Bonnici, and Maguire 2014). Interestingly, lithium users showed greater preservation of CA3 volume compared to lithium nonusers (*n* = 464) (Haukvik et al. 2022). Reductions in hippocampal volume may be explained by reductions in parvalbumin‐positive interneuron number and size (Zhang and Reynolds 2002; Konradi et al. 2010), which may impact the regulation of the hyperexcitable fraction of pyramidal cells in the CA3.

Recent advances in stem cell technology have allowed for the precise investigation of the properties of patient‐derived in vitro models of CA3 pyramidal cells to further elucidate potential cellular‐level abnormalities in BD (Mertens et al. 2015). Specifically, to study the electrophysiological properties associated with lithium responsiveness, patient‐derived cells have been reprogrammed into induced pluripotent stem cells (iPSCs) and subsequently differentiated into CA3 pyramidal cell–like neurons (CA3‐PCs) (Stern, Sarkar, Stern, et al. 2020). Notably, CA3‐PCs derived from lithium responders are hyperexcitable, which is normalized upon lithium exposure (Stern, Sarkar, Stern, et al. 2020). This phenomenon is absent in CA3‐PCs from both healthy controls and lithium nonresponders (Stern, Sarkar, Stern, et al. 2020). Yet, CA3‐PCs derived from lithium nonresponders have exhibited a high diversity of activity, with a mixed population of hyperexcitable and hypoexcitable cells (Stern, Sarkar, Galore, et al. 2020). This electrophysiological diversity is a distinct abnormality between lithium responders and nonresponders and may be a potential key to understanding the neural underpinnings of cognitive dysfunction in lithium‐nonresponsive BD.

Together, there is genetic, structural, and cellular electrophysiological evidence suggesting that CA3 structure and functioning are likely to be abnormal in BD. However, to link these abnormalities to observable behaviors, we must understand (A) the computations carried out by the CA3 circuit, (B) how these computations are affected by the neurobiological abnormalities observed in BD, and (C) how these computations connect to observable behaviors. As a first step, we must understand how variability in cellular excitability in the CA3 relates to circuit‐level computations. Therefore, in this study, we leverage computational modeling to examine how the diversity of excitability in CA3‐PCs might affect pattern completion in the CA3. This work will facilitate our ability to bridge the gap between cellular properties and network‐level function in BD, providing more specific predictions about the memory dysfunctions seen in lithium‐nonresponsive BD.

## 2 Methods

### 2.1 Computational Model

**FIGURE 1:**
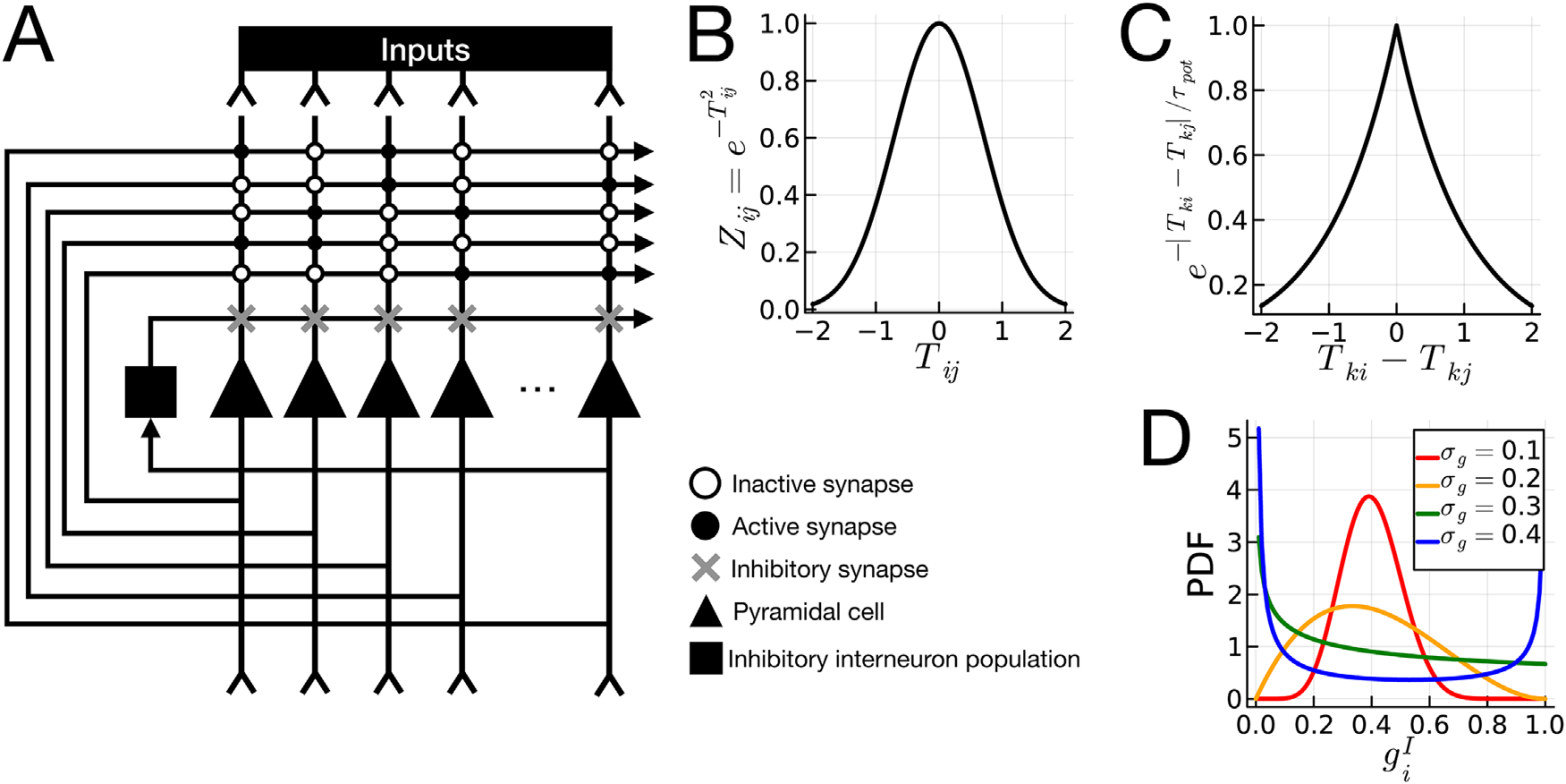
Illustration of the computational model. (A) Model architecture. Solid triangles represent the CA3 pyramidal cells. Solid square is the inhibitory interneuron population. Solid circles are active synapses at which plasticity occurs. Open circles are inactive synapses, which effectively represent no connection or plasticity between two neurons. Gray “X” markers are inhibitory synapses. Note that while there are inhibitory inputs to all pyramidal cells, they will vary in strength depending on the value of *g_i_^I^*. (B) Relationship between spike time *T_ij_* and activation level *Z_ij_*. (C) Symmetric spike timing–dependent plasticity function (Mishra et al. 2016; Rebola, Carta, and Mulle 2017). The *x*‐axis plots the difference in spike time between neurons *i* and *j* during pattern *k*, denoted *T_ki_*—*T_kj_*, and the *y*‐axis denotes the resulting degree of synaptic potentiation, exp{‐|*T_ki_*—*T_kj_* |/τ_pot_}, which applies only at synapses that are connected (i.e. “active synapses”). (D) Illustration of Beta distribution with *µ_g_* = 0.4, and *σ_g_* ∈ {0.1, 0.2, 0.3, 0.4}. The *x*‐axis shows the value of *g_i_^I^*, and the *y*‐axis is the probability density.

Our study extends a previously developed model of the hippocampal CA3 for large‐scale simulations (Gibson and Robinson 1992; Bennett, Gibson, and Robinson 1994; Guzman et al. 2016; Mishra et al. 2016). We use an implementation with *n* = 3000 integrate‐and‐fire‐type glutamatergic neurons and a pooled inhibitory population modeled as a single unit. The model architecture is illustrated in Figure 1, with detailed mathematical descriptions in the Supporting Information. Simulations were conducted in the Julia programming language (v. 1.9.0), with code on GitHub. Here, we provide an overview of the model and experiments, focusing on higher level concepts and their connection to the relevant translational neuroscience.

CA3 pyramidal cells are modeled as integrate‐and‐fire neurons, where presynaptic input strengths are modeled as “weights.” When the total weighted sum of presynaptic activity exceeds the neuron's depolarization threshold, the postsynaptic neuron fires an action potential. This simplified model allows us to (A) simulate larger networks and (B) study the effects of narrowly defined features of a neural network while limiting the noise induced by having too many other parameters to control. Given that our focus is the diversity of excitability of glutamatergic neurons, the simple integrate‐and‐fire type neuron model is suitable since the diversity of excitability can be simply controlled by altering the variance of firing thresholds across neurons.

As with previous implementations of this standard model of the CA3 (Gibson and Robinson 1992; Bennett, Gibson, and Robinson 1994; Guzman et al. 2016; Mishra et al. 2016), we model the GABAergic interneurons as a pooled inhibitory population that receives input from all CA3 pyramidal cells, then projects some level of inhibition back, depending on the strength of GABAergic synapses at the postsynaptic CA3 pyramidal cell. Each CA3 pyramidal cell, indexed as *i* = {1, 2, …, *n*}, has its own GABAergic synaptic input strength *g_i_^I^*, sampled from a Beta distribution (Figure 1D) with mean *µ_g_* and standard deviation *σ_g_*. Higher *µ_g_* means higher average inhibition, resulting in more sparse pyramidal cell activity, and higher 𝜎*_g_* means higher diversity of excitability. In this paper, to model the varying degrees of diversity in CA3 pyramidal cell excitability, as observed in BD, we manipulated *σ_g_*. That is, since the CA3 pyramidal cells of lithium nonresponders have been shown to have more diverse levels of excitability than those from lithium responders, then a CA3 model corresponding to lithium nonresponders would have a higher 𝜎*_g_* than a network that models the CA3 of lithium responders.

Excitatory connections between CA3 pyramidal cells, known as *recurrent collaterals* (Rebola, Carta, and Mulle 2017), putatively facilitate memory storage and pattern completion in the CA3 (Marr 1971; Bennett, Gibson, and Robinson 1994) by creating an *autoassociative attractor network* (Marr 1971; McNaughton and Morris 1987). That is, the co‐activation of neurons representing a memory strengthens synapses between them, meaning that during retrieval, activation of one neuron will reactivate the others, completing the memory. To this end, the ability of the CA3 attractor network to encode and retrieve memories depends on connectivity and plasticity at the CA3 recurrent collaterals. Traditional models assume that all CA3 pyramidal cells are connected (Hopfield 1982; Krotov and Hopfield 2021), which is biologically implausible. We modeled connectivity between pyramidal cells as in Mishra et al. (2016), with neurons connected with a fixed probability c*. All experiments were repeated across different connectivity probabilities as sensitivity analyses (25%, 50%, and 75%). Learning was modeled using symmetrical spike timing–dependent plasticity (which is observed in CA3 recurrent collateral synapses (Mishra et al. 2016; Rebola, Carta, and Mulle 2017); see Figure 1C), whereby if a presynaptic and postsynaptic neuron fire closely together in time, the connection between them will strengthen. Longer intervals between pre‐ and postsynaptic spikes correspond with less synaptic strengthening.

### 2.2 Encoding Memories in the CA3 Network Model

In this paper, a “pattern” or “memory” is a set of neuronal activations stored in the CA3. A pattern is modeled by stimulating a subset of neurons to fire at randomly chosen times. Neurons that fire closely together in time will strengthen their recurrent collateral synapses via the symmetric spike timing–dependent plasticity mechanism described above. Different patterns are simulated by selecting different neuronal subsets and firing times. Increasing the number of patterns stored in each CA3 network will increase pattern completion difficulty due to memory interference.

For each experiment, we encoded *m* different patterns in the CA3 model (specifically 5, 10, 25, or 50) while varying the proportion of active neurons, *a* (*a* equals to 0.01, 0.05, 0.1, and 0.2), as a sensitivity analysis to examine the degree to which the effects of diverse excitability were affected by storage load and pattern sparsity.

### 2.3 Retrieval of Patterns in the CA3 Network Model

After encoding, we evaluate pattern completion by presenting the network with partial “retrieval cues,” created by activating only 50% of the neurons that were active during the storage of a particular pattern (Mishra et al. 2016). The accuracy of pattern completion was then evaluated by examining the Pearson correlation between the recovered and original patterns.

### 2.4 Experiments

We examined pattern completion accuracy in the CA3 under different degrees of variability of pyramidal cell excitability. The primary outcome was pattern completion accuracy, measured using the Pearson correlation, denoted 𝜌(*X, Z*), between the recovered pattern vector *X =* (*X_1_, X_2_,…, X_j_, …, X_n_*) and its corresponding ground truth pattern vector *Z =* (*Z_1_, Z_2_,…, Z_j_, …, Z_n_*), where *X_j_* and *Z_j_* are continuous values representing the activity levels of neuron *j* in the recovered and ground truth patterns, respectively. A value of *X_j_* > 0 means that neuron *j* was active in the final retrieved pattern. A value of *Z_j_* > 0 means that neuron *j* was active in the actual ground truth pattern encoded in the network.

As secondary outcomes, we examine the amount of valid and spurious activity during recall. Valid activity is computed as the *hit rate*, denoted *H*, which is the probability that a neuron would be correctly active in the retrieved pattern when it was also active in the originally encoded “ground truth” pattern. Spurious activity is computed as the *false alarm rate*, which is the probability that a neuron was inappropriately activated during retrieval when, in fact, it was not active as part of the originally encoded ground truth pattern.

Our independent variable of interest is the diversity of pyramidal cell activity. Recall that this is modeled by changing the parameter *σ_g_* that governs the variability of inhibition sensitivity across pyramidal cell neurons in the model. Larger values of *σ_g_* simulate the greater levels of heterogeneous excitability, which have been experimentally observed in iPSC models of lithium nonresponsive BD (Stern, Sarkar, Galor, et al. 2020). In the present study, we examined the pattern completion ability of the network while systematically varying *σ_g_* from a value of 0 (meaning completely homogeneous excitability levels) to a maximal value of *σ_g_* ^max^ = sqrt[*µ_g_* (1 − *µ_g_*)] (which is the maximal amount of excitability variability allowed for a given mean of *µ_g_*). We therefore repeated all analyses under different levels of mean inhibition sensitivity *µ_g_* (specifically 0.1, 0.2, 0.3, and 0.4). All experiments were also repeated under different values of pattern load *m*, pattern sparsity *a*, and network connectivity probability c* (each range was previously stated above).

The effect of *σ_g_* on pattern completion performance was quantified using linear regression of *ρ* against *σ_g_*, with *m*, *µ_g_*, *a*, and c* as covariates. We assumed a statistical significance threshold of *ɑ* = 0.05.

Following our primary analysis, we probed the degree to which pattern completion errors were attributable to the hyperexcitable versus hypoexcitable fraction of pyramidal cells. This follows from the fact that for a given amount of variance in the distribution of inhibition sensitivity values *g_i_^I^*, there will be some pyramidal cells that have higher than average levels of sensitivity to inhibition (i.e., those neurons *i* such that *g_i_^I^* > *µ_g_*, which we call the “hypoexcitable fraction”) and some that have lower than average levels of sensitivity to inhibition (i.e., those neurons *i* such that *g_i_^I^* < *µ_g_*, which we call the “hyperexcitable fraction”). Across multiple average levels of inhibition (*µ_g_* ∈ {0.1, 0.2, 0.3, 0.4, 0.5, 0.6}) and variability of pyramidal cell excitability (*σ_g_* = 0.01 to *σ*_g_^max^ in increments of 0.02, with *σ_g_* ^max^ = sqrt(*µ_g_* (1 − *µ_g_*))), we examined the pattern completion error rate in the hypoexcitable and hyperexcitable fractions, respectively, to determine whether errors were systematically generated by the hyperexcitability or hypoexcitability of neurons in a diverse population.

## 3 Results

### 3.1 Both Hyperexcitability and Diverse Excitability Impair Pattern Completion

**FIGURE 2:**
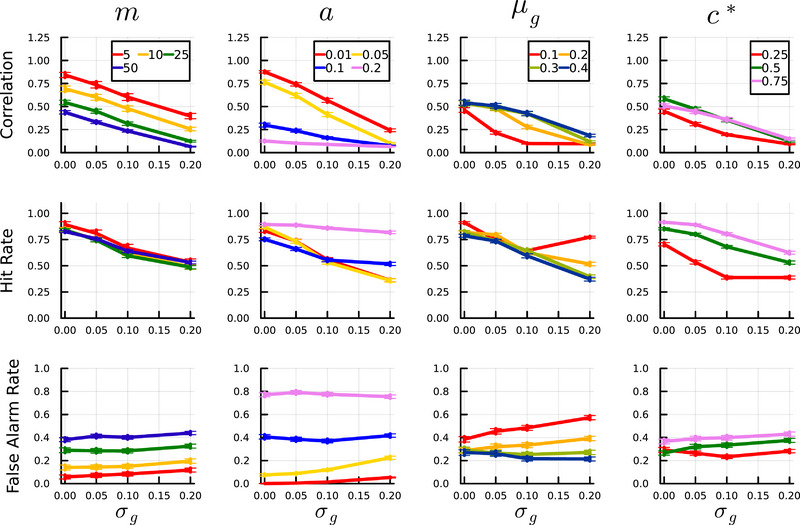
Effects of variability in pyramidal cell activity (*σ_g_*) on pattern completion performance, measured by the Pearson correlation between the true and recovered patterns. The top row of plots shows correlation results, while the second and third rows show the hit rates and false alarm rates with respect to *σ_g_*. Columns display results with respect to various moderating factors, including the number of encoded patterns (*m*), the proportion of neurons active for each pattern (*a*), the mean level of inhibition (*µ_g_*), and average connectivity (c*).

Table 1 and Figure 2 show the effects of hyperexcitability and diversity thereof on pattern completion performance in our CA3 model. Diverse pyramidal cell excitability, measured by *σ_g_*, reduced pattern completion performance in all cases (top row of plots in Figure 2; *β* = −2.00, *SE* = 0.03, *p* < 0.001, Table 1). The effect was invariant to the number of patterns stored in the network (*m*), evinced by the relatively parallel decline in correlation across values of *m* in Figure 2. Overall hyperexcitability, captured by either (A) higher values of *a* (the proportion of neurons active in each pattern; *β* = −2.70, *SE* = 0.03, *p* < 0.001) or (B) lower values of *µ_g_* (*β* = 0.72, *SE* = 0.02, *p* < 0.001), was associated with worse pattern completion performance. Hyperexcitability impaired pattern completion performance primarily by increasing false alarm rates (i.e., increasing the number of spuriously active neurons), while diversity of excitability (*σ_g_*) impaired pattern completion largely by reducing hit rates (Figure 2). The impact of excitability diversity *σ_g_* was independent of overall levels of inhibition *µ_g_* or the proportion of neurons active in each pattern (*a*), suggesting that correction of overall levels of excitability is insufficient to entirely correct pattern completion deficits in the presence of variable excitability of CA3 pyramidal cells (Figures S1–S3).

**TABLE 1:** Ordinary least squares estimates of effects of diversity of hyperexcitability (*σ_g_*), as well as overall levels of excitability (the proportion of neurons active for each pattern, also called the “pattern density” *a*, and the average inhibitory strength across neurons, *µ_g_*), controlled for the overall pattern load (*m*) and connectivity rate (c*).

	*β*	*SE*	*t*	*p*	95% CI low	95% CI high
Intercept	0.73	0.01	86.51	< 0.001	0.72	0.75
Pattern load (*m*)	−0.01	0.00	−52.19	< 0.001	−0.01	−0.01
Pattern density (*a*)	−2.70	0.03	−100.36	< 0.001	−2.75	−2.65
Connectivity probability (c*)	0.13	0.01	13.99	< 0.001	0.11	0.15
Mean inhibition level (*𝜇_g_*)	0.72	0.02	41.31	< 0.001	0.68	0.75
Variability of excitability (*σ_g_*)	−2.00	0.03	−77.12	< 0.001	−2.05	−1.95

Abbreviations: CI, confidence interval; *p*, *p*‐value; *SE*, standard error; *t*, *t*‐statistic; *β*, regression coefficient.

### 3.2 Variable Excitability Induces Pattern Completion Errors Through the Hyperexcitable Cells

**FIGURE 3:**
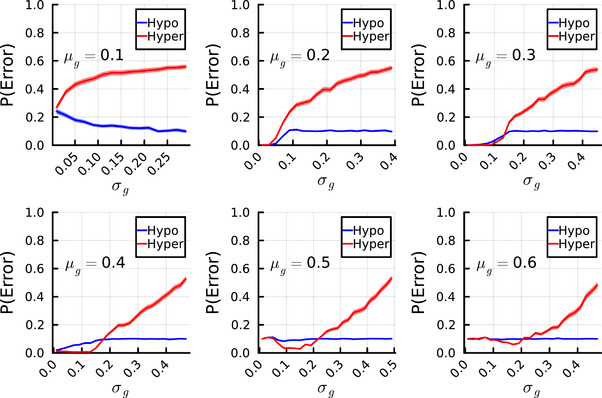
Pattern completion error rates (*y*‐axes) in hyperexcitable (red lines; inhibition level less than mean *µ_g_*) and hypoexcitable (blue lines; inhibition level less higher than mean *µ_g_*) neurons, in relation to the level of variability in pyramidal cell activity (*x*‐axes; *σ_g_*). Each plot corresponds to a specific mean level of inhibition (*µ_g_*). Solid lines are mean error rates, and ribbons are 95% confidence intervals.

Figure 3 shows the results of evaluating error rates in pattern completion in relation to the variability of excitability levels (*σ_g_*) across different levels of mean inhibition in the network (*µ_g_*). Across all mean inhibition levels, increases in variability of excitability resulted in higher pattern completion error rates primarily attributable to the hyperexcitable half of the CA3 pyramidal cell population. At low levels of inhibition (*µ_g_* = 0.1; high global levels of hyperexcitability), the lower bound on error rates is approximately 0.25. The lower bound on error rate subsequently decreases to almost 0 as the overall excitability levels decline. However, if overall network excitability is overcorrected via higher levels of inhibition (*µ_g_* above 0.4), we observe that the lower bound of pattern completion error increases, suggesting that excessive correction of the mean excitability levels in the CA3 network may also impair pattern completion. At high levels of inhibition, the pattern completion error rate was constant for the hypoexcitable fraction of pyramidal cells.

## 4 Discussion

We showed that variability of CA3 pyramidal cell excitability, observed in iPSC‐derived CA3 pyramidal cell–like neurons from lithium nonresponders (Stern, Sarkar, Galor, et al. 2020), impairs pattern completion in a CA3 network model. This impairment was independent of average population‐level excitability and all other network parameters, suggesting that variability in iPSC‐derived pyramidal cell activity is potentially a candidate independent marker of episodic memory impairments in lithium nonresponders. Pattern completion errors arose primarily from the pyramidal cells with higher‐than‐average excitability. While this might suggest that minimizing neuronal excitability is the solution to this problem, we observed that under some circumstances (i.e., when the overall average excitability levels are low), pattern completion errors arise primarily from hypoexcitable neurons relative to the rest of the population. Our findings suggest that cognitive impairments in lithium nonresponders may differ physiologically from those in lithium responders, necessitating different approaches to remedy episodic memory deficits in these groups.

The 50% most excitable neurons (i.e., the hyperexcitable fraction) substantially contributed to pattern completion errors. However, at low overall excitability levels (high inhibition, high *µ_g_*), errors increased due to the 50% least excitable neurons. This suggests that using hyperexcitability‐lowering mood stabilizers in lithium nonresponders (Mertens et al. 2015; Santos et al. 2021) may iatrogenically create a ceiling effect on their cognitive performance. Therefore, we must understand how mood stabilizers may *normalize the distribution of neuronal excitability*, rather than merely reducing hyperexcitability overall. The complexity of this type of intervention stands in contrast to the potential effects of treatment on lithium responders, whose CA3 pyramidal cells do not show a wide variation in hyperexcitability levels (Stern, Sarkar, Galor, et al. 2020). For lithium responders, simply limiting cellular hyperexcitability would be predicted to improve pattern completion. Future studies should examine behavioral measures of pattern completion in lithium responders and nonresponders from whom iPSC‐derived CA3 pyramidal cell–like neurons have been cultured and whose excitability distributions (mean excitability and variance) are well characterized. Such studies should then characterize how neuronal excitability distributions are affected when the neuronal cultures are exposed to mood stabilizers to which patients demonstrably respond or fail to respond, clinically. Our results predict that persistent variance in excitability despite treatment with excitability‐lowering mood stabilizers will be associated with worse behavioral pattern completion performance in patients with BD.

Our results highlight the importance of understanding the diversity of neuronal excitability in lithium nonresponders. Stern et al. (Stern, Sarkar, Stern, et al. 2020; Stern, Sarkar, Galor, et al. 2020; Stern et al. 2018) showed that BD nonresponders have reduced sodium currents and increased potassium currents compared to healthy controls. Differences in sodium currents were found to potentially mediate variation between hyperexcitable and hypoexcitable neurons from lithium nonresponders, where the neurons with average or high sodium currents were mostly hyperexcitable, while the neurons with sodium currents below the average (which is already lower in the nonresponders compared to both controls and the responders) were mostly hypoexcitable and unable to produce action potentials even in response to current injections. We believe that this reduction in the sodium currents paired with an increase in potassium currents may strongly influence BD nonresponders' neuronal electrophysiological diversity. However, the underlying cause of this diversity in sodium and potassium conductances within individual patients is unknown. Future experimental work with iPSC models of lithium nonresponders is required to answer these questions.

Chronic lithium treatment increased sodium currents and decreased potassium currents in neurons derived from BD lithium, “normalizing” their neurophysiology to better resemble control neurons (Stern, Sarkar, Stern, et al. 2020; Stern et al. 2018). In contrast, valproic acid (Santos et al. 2021) reduced sodium currents in neurons from both responders and nonresponders, driving BD nonresponder‐derived neurons even *further* away from normal neurophysiology (Tripathi et al. 2023), further supporting our study's prediction that using medication to simply decrease hyperexcitability may not be the optimal treatment. The design of novel mood‐stabilizing therapies using iPSC‐derived neuron models should therefore consider medications’ effects on both single neuron physiology *and* neurons’ behavior as populations in a network.

A major strength of our study is the simplified and well‐controlled model of the CA3, allowing precise control over excitability variance in the CA3 pyramidal cell population. This simplicity facilitated the control of many potential confounding factors, demonstrating that excitability variance and pattern completion deficits were unaffected by overall inhibition, connectivity, pattern load, and network sparsity. However, the simplicity is also a limitation, given that the model abstracts away many details, including the diversity of interneuron types (Neymotin et al. 2011) and structural/biophysical properties of the pyramidal cell somatodendritic tree (Bennett and Lagopoulos 2014; Szymanski and Minichiello 2022). Although we could not identify a specific level of experimentally determined variance in CA3 pyramidal cell–like excitability, our results are robust to this because we examined pattern completion performance across the full range of excitability variance available under our model. Our model also employs a relatively simple and dense connectivity pattern between CA3 pyramidal cells. The connectivity patterns between CA3 pyramidal cells have previously been shown to incorporate complex motifs (Guzman et al. 2016), although simpler dense and random connectivity patterns as employed in the present study have been shown to generate similar network behavior (Mishra et al. 2016). To efficiently use computational resources, we employed the simpler approach, which facilitated our examination of different network conditions in sensitivity analyses.

In conclusion, our study suggests that the diverse excitability of CA3 pyramidal cell–like neurons observed in lithium nonresponders may predict pattern completion deficits. These deficits are invariant to overall excitability levels, suggesting that they may persist even if overall CA3 pyramidal cell excitability is controlled. These predictions should be validated experimentally using behavioral pattern completion paradigms that require patients to first encode a set of stimuli, after which they must either (A) recall those stimuli or (B) discriminate the studied stimuli from novel/lure stimuli, given partial or noisy/corrupted cues (Liu et al. 2016). Furthermore, it would be useful to examine these behavioral pattern completion deficits and how they correlate to clinical and cellular responses to lithium or anticonvulsant mood stabilizers. Our results suggest that without normalizing the complete distribution of cellular excitability in the CA3, pattern completion deficits may persist in patients with BD.

## Author Contributions

**Abraham Nunes**: conceptualization, validation, software, formal analysis, data curation, writing–original draft, writing–review and editing, resources, project administration, visualization, funding acquisition, investigation, methodology. **Selena Singh**: writing–review and editing. **Anouar Khayachi**: writing–review and editing. **Shani Stern**: writing–review and editing. **Thomas Trappenberg**: supervision, writing–review and editing. **Martin Alda**: writing–review and editing, supervision.

## Ethics Statement

The authors have nothing to report.

## Conflicts of Interest

The authors declare no conflicts of interest.

### Peer Review

The peer review history for this article is available at https://publons.com/publon/10.1002/brb3.70209

## Supporting information



Supplementary Information

## Data Availability

The data that support the findings of this study are openly available in GitHub at https://github.com/cpsylab/CA3‐Diversity‐Pattern‐Completion.
