# Supplementary material for: The Impact of Electrophysiological Diversity on Pattern Completion in Lithium Nonresponsive Bipolar Disorder: A Computational Modeling Approach
Source: Brain Behav. 2025 Jan 20;15(1):e70209. doi: 10.1002/brb3.70209 (PMC11745123; doi:10.1002/brb3.70209)
Supplement: Supplementary file 1 — Supplementary Information [file BRB3-15-e70209-s001.pdf]

Supplementary Materials for *The Impact of Electrophysiological Diversity on Pattern Completion in Lithium Nonresponsive Bipolar Disorder: A Computational Modelling Approach*

Abraham Nunes [1,2,*], Selena Singh [3], Anouar Khayachi [4], Shani Stern [5], Thomas Trappenberg [2], Martin Alda [1]

[1] Department of Psychiatry, Dalhousie University, Halifax, Nova Scotia, Canada
[2] Faculty of Computer Science, Dalhousie University, Halifax, Nova Scotia, Canada
[3] Department of Psychology, Neuroscience & Behaviour, McMaster University, Hamilton, Ontario, Canada
[4] Montreal Neurological Institute, Department of Neurology & Neurosurgery, McGill University, Montreal, Quebec, Canada
[5] Sagol Department of Neurobiology, Faculty of Natural Sciences, University of Haifa, Israel

Contents

Supplementary Methods **2**
Encoding Phase and Learning in the Model 2
Pattern Completion Task and Recall Dynamics in the Model 2
Experiments 3
Supplementary Results **4**
References **7**

Supplementary Methods

Encoding Phase and Learning in the Model

For a given storage cycle, a pattern $i \in \{1,2,\ldots,m\}$ is presented to the CA3. Each pattern is characterized by activity in $n_a = \lfloor n \times a \rfloor$ neurons, where $a \in [0, 1]$ is the proportion of neurons active in a given pattern, and $\lfloor x \rfloor$ is the floor function (that rounds down).

For each pattern $i \in \{1,2,\ldots,m\}$ presented to the CA3 in a given storage cycle, let $T_{ij} \sim Normal(0, s_T)$ be the spiking time of neuron $j \in \{1,2,\ldots,n\}$, with mean 0, and standard deviation $s_T > 0$. Following Mishra et al. (1) we set $s_T = 0.2$, corresponding to 20% of a storage cycle. The activation level of neuron j in pattern i is $Z_{ij} = \exp\{-T_{ij}^2\}$ (Figure 1). For the excitatory synapse from neuron j to i, plasticity during the encoding phase was implemented using the following symmetric spike timing-dependent plasticity rule (1):

$$J_{ij} = \epsilon_{ij} \times \left(J^o + \sum_{k=1}^{m} \delta_{ijk}\left[exp\left\{-\left|T_{ki} - T_{kj}\right|/\tau_{pot}\right\}\right]_0^1\right),$$

where $\epsilon_{ij} \sim Bernoulli(c_{ij})$ is a binary random variable with probability c_{ij} indicating whether there is an excitatory connection from neuron j to i, δ_{ijk} is an indicator variable denoting whether both neurons i and j are active during pattern k, and $[x]_0^1 = max\{min\{x, 1\}, 0\}$. The value $J^o = 0$ indicates that the base synaptic strength is 0. The synaptic plasticity time constant τ_{pot} was set to 1, corresponding to a single storage cycle (1). In the present model, we assume a random (Erdős–Rényi) connectivity structure with probability $c_{ij} = c^*$.

Pattern Completion Task and Recall Dynamics in the Model

After a CA3 network learns patterns $Z = \{ Z_{ij} : i \in 1 \ldots m, j \in 1 \ldots n\}$, we evaluated pattern completion by presenting the network with an incomplete/corrupted version of each pattern in Z, and evaluating the accuracy with which the CA3 network could recover the original pattern.

Dynamics of pattern completion during recall were simulated by feeding a partial or noisy "seed" input pattern to the CA3 network, and allowing network dynamics to equilibrate over 10 recall cycles. The seed pattern corresponding to pattern $k = 1,2,\ldots,m$ is controlled by parameters, $b_1 \in [0, 1]$ (the proportion of neurons in the seed pattern that correspond to valid activations in pattern k) and $b_2 \in [0, 1]$ (the proportion of spurious additional activations that were not present in the true pattern k). Increasing b_1 corresponds to providing the network with a seed pattern that is more accurately reflective of a true pattern upon which the synapses were trained during encoding. Increasing b_2 corresponds to adding more "noise" in the form of spuriously active neurons. In the present study, as in Mishra et al., (1) we set $b_1 = 0.5$, and $b_2 = 0$.

The total synaptic input into neuron i at recall cycle t is:

$$h_i(t) = n^{-1} \sum_{j=1}^{n} J_{ij}\, \Theta\left(t - T_j\right) exp\left\{-\left(t - T_j\right)/\tau_m\right\},$$

where $\Theta(x)$ is the Heaviside step function, which takes a value of 1 for $x > 0$, and 0 otherwise, τ_m is the time constant for neuron i's membrane potential (τ_m is assumed to be the same for all pyramidal cells in the model), and T_j is the spike time of neuron j at the previous recall cycle. Neuron i spikes as part of pattern $k \in \{1, 2, \ldots, m\}$ if $h_i(t) > g_i^o + g_i^I S$, where S is the sum of all neural activity at the previous recall cycle, $g_i^o = 0$ is the basal action potential threshold for

neuron i, and g_i^I is a factor scaling the degree of inhibition received by neuron i. S models a general inhibitory population whose activity is proportional to the total network activity at the previous recall cycle. All simulations were conducted using custom scripts in the Julia programming language (v. 1.9.0), which are available on GitHub.

Experiments

We are interested in examining pattern completion performance in the CA3 under conditions of different degrees and variability of pyramidal cell excitability. Specifically, for a given CA3 network configuration, we measure pattern completion performance as the Pearson correlation between the recovered pattern X_i and its corresponding ground truth pattern Z_i. We denote the Pearson correlation as $\varrho(X_i, Z_i)$, where $X_i = (X_{i1}, X_{i2}, ..., X_{in})^{\mathrm{T}}$ is a vector corresponding to the final activity level of each neuron after completion of pattern i, and $Z_i = (Z_{i1}, Z_{i2}, ..., Z_{in})^{\mathrm{T}}$ is the true activity of each neuron for pattern i. As secondary outcomes, we examine the amount of valid and spurious activity during recall. Valid activity is computed as the *hit rate*

$$H_i = (\Sigma_{j=1,\ldots,n}\, \mathbb{1}[X_{ij} > 0]\, \mathbb{1}[Z_{ij} > 0])/(\Sigma_{k=1,\ldots,n}\, \mathbb{1}[Z_{ik} > 0])$$

where $\mathbb{1}[x]$ is an indicator function taking values 1 if x is true, and 0 otherwise. Spurious activity is computed as the *false alarm rate*

$$FA_i = (\Sigma_{j=1,\ldots,n}\, \mathbb{1}[X_{ij} > 0]\, \mathbb{1}[Z_{ij} = 0])/(\Sigma_{k=1,\ldots,n}\, \mathbb{1}[Z_{ik} = 0])$$

which is the propensity for neuron j to be active during recall, despite not being involved in the encoded pattern Z_i.

Our independent variable of interest is the standard deviation of the inhibitory scaling factor g_i^I, denoted σ_g. A wider distribution on g_i^I simulates the heterogeneous excitability observed in lithium nonresponsive BD (2,3). The values g_i^I are sampled from a Beta distribution with mean μ_g and standard deviation σ_g, where $\sigma_g^{\,2} < \mu_g\,(1\text{-}\mu_g)$. We examine the pattern completion ability of the network while systematically varying σ_g, conditional upon m, μ_g, a, and c^* for $i,j = 1,2,\ldots,n$. We measured the effect of σ_g on pattern completion performance using linear regression of ϱ against σ_g, with m, μ_g, a, and c^* as covariates.

We then probed the degree to which pattern completion errors were attributable to the hyperexcitable vs. hypoexcitable fraction of pyramidal cells. Across multiple average levels of inhibition ($\mu_g \in \{0.1, 0.2, 0.3, 0.4, 0.5, 0.6\}$), we examined the pattern completion error rate in relation to pyramidal cell inhibition variance ($\sigma_g = 0.01$ to $\sigma_g^{\max}$ in increments of 0.02, with $\sigma_g^{\max} = \mathrm{sqrt}(\mu_g(1\text{-}\mu_g))$).

Supplementary Results

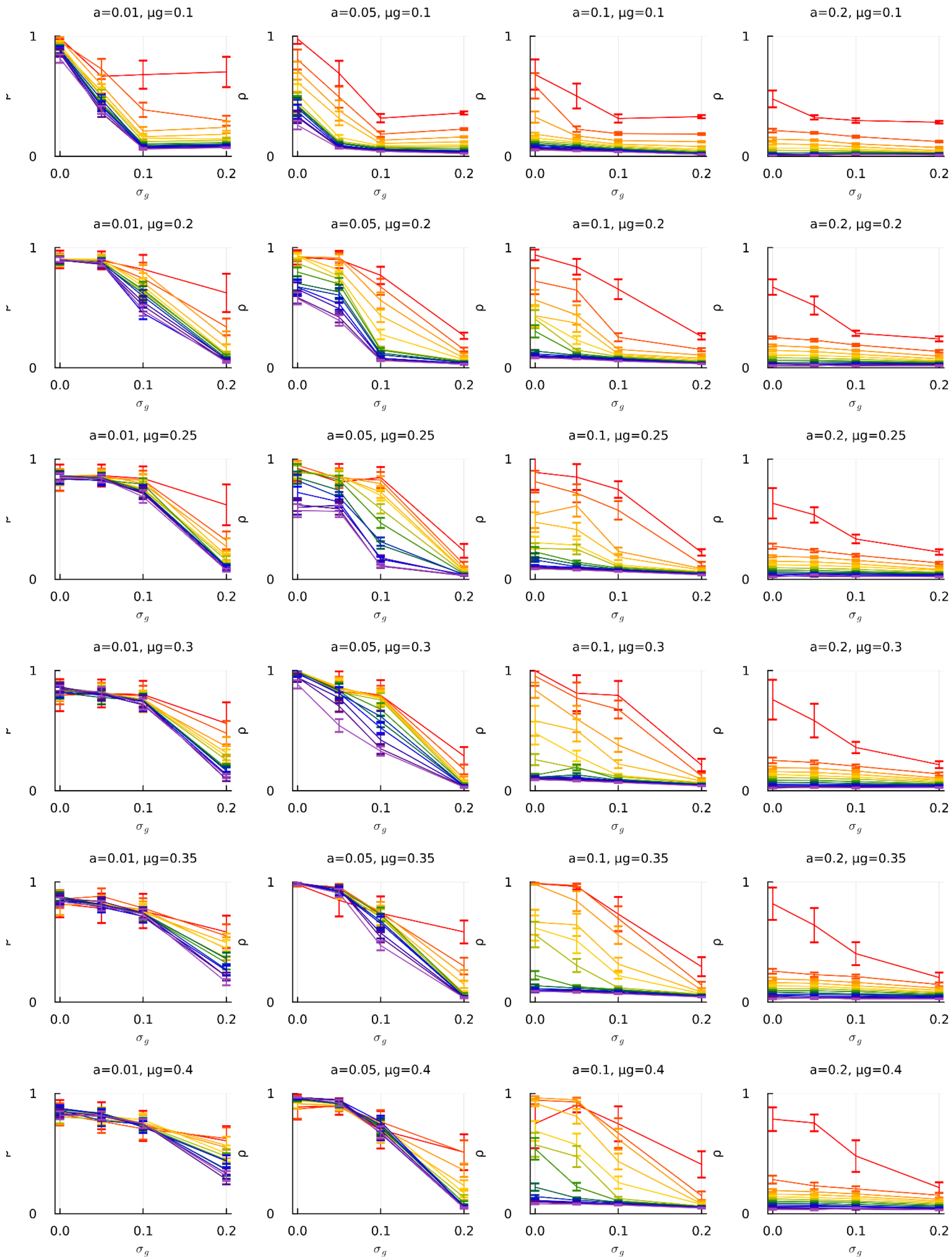

Figure S1. Activity correlation (y-axis) and interactions between the diversity of hyperexcitability (σ_g; x-axis), activation level (a), and mean level of inhibition (μ_g). Coloured lines represent the pattern load, ranging from m=5 (red) to m=65 (indigo), in steps of 5, according to a rainbow palette.

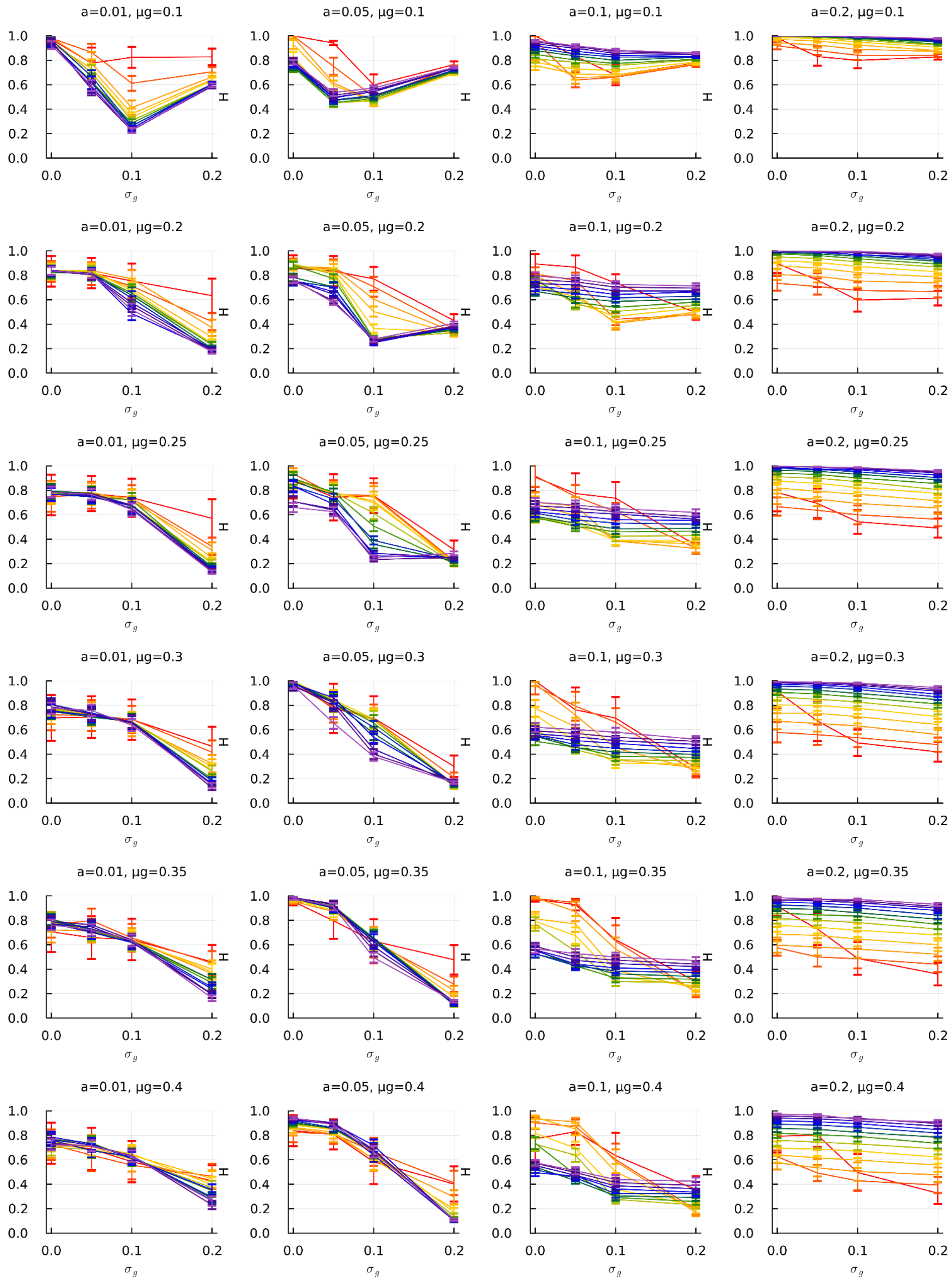

Figure S2. Hit rates (y-axis) and interactions between the diversity of hyperexcitability (σ_g; x-axis), activation level (a), and mean level of inhibition (μ_g). Coloured lines represent the pattern load, ranging from m=5 (red) to m=65 (indigo), in steps of 5, according to a rainbow palette.

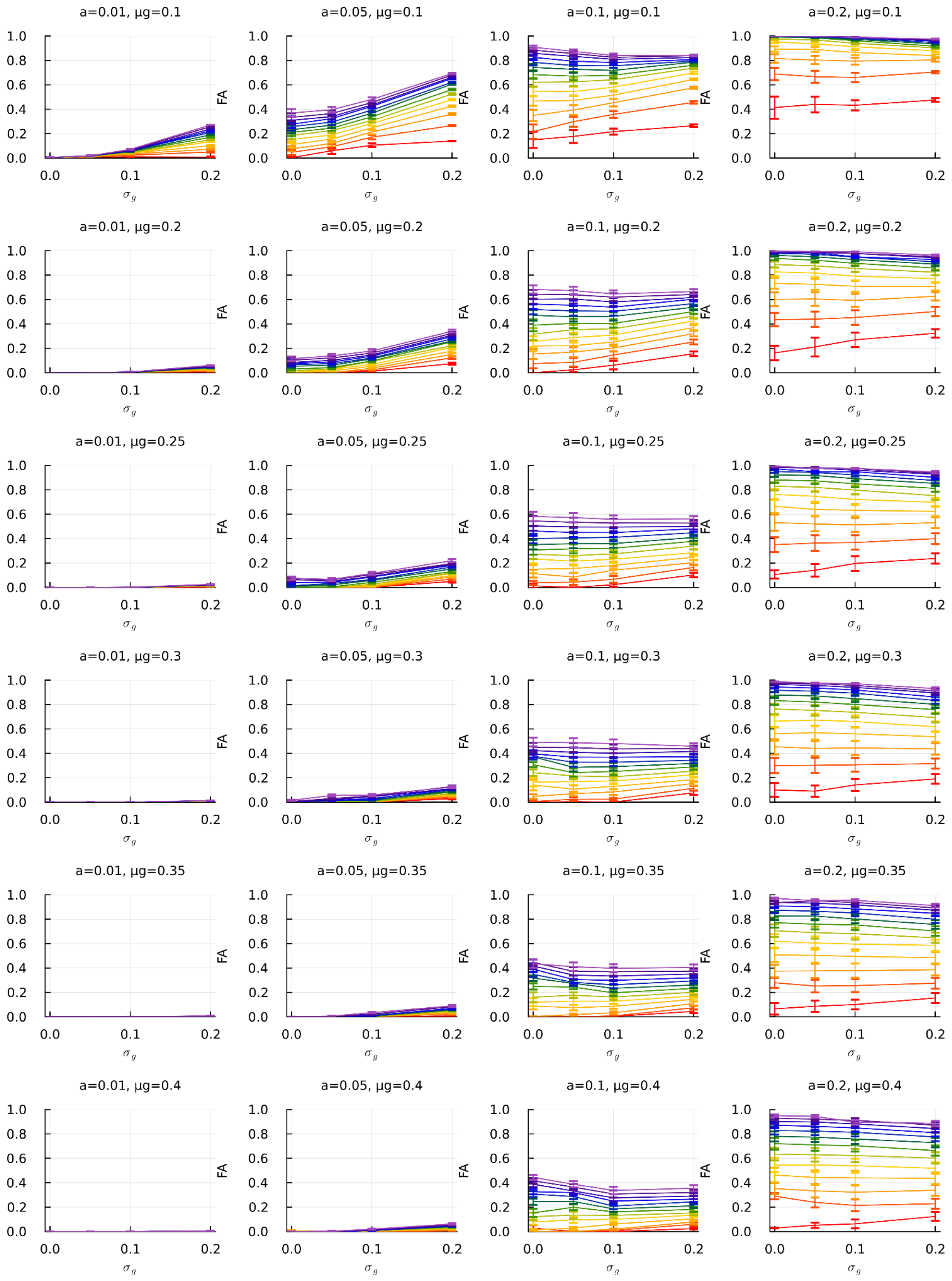

Figure S3. False alarm rates (y-axis) and interactions between the diversity of hyperexcitability (σ_g; x-axis), activation level (a), and mean level of inhibition (μ_g). Coloured lines represent the pattern load, ranging from m=5 (red) to m=65 (indigo), in steps of 5, according to a rainbow palette.

References

1. Mishra RK, Kim S, Guzman SJ, Jonas P. Symmetric spike timing-dependent plasticity at CA3–CA3 synapses optimizes storage and recall in autoassociative networks. Nat Commun. 2016 Sep;7(1):11552.
2. Stern S, Sarkar A, Stern T, Mei A, Mendes APD, Stern Y, et al. Mechanisms Underlying the Hyperexcitability of CA3 and Dentate Gyrus Hippocampal Neurons Derived From Patients With Bipolar Disorder. Biological Psychiatry. 2020 Jul;88(2):139–49.
3. Stern S, Sarkar A, Galor D, Stern T, Mei A, Stern Y, et al. A Physiological Instability Displayed in Hippocampal Neurons Derived From Lithium-Nonresponsive Bipolar Disorder Patients. Biological Psychiatry. 2020 Jul;88(2):150–8.
